# Frogs have layers: a comparative histological study of colour polymorphism in *Oophaga* poison frogs

**DOI:** 10.7717/peerj.21696

**Published:** 2026-09-16

**Authors:** Vasiliki Mantzana Oikonomaki, Mats Johannes Wiborg, Giselle Tamayo-Castillo, Roberto Ibáñez, Ariel Rodríguez, Heike Pröhl

**Affiliations:** 1 Institute of Zoology, Tierärztliche Hochschule Hannover, Hannover, Germany; 2 Centro de Investigaciones en Productos Naturales (CIPRONA) & Escuela de Química, Universidad de Costa Rica, San Jose, Costa Rica; 3 Smithsonian Tropical Research Institute, Panama, Panama

**Keywords:** Aposematic, Cryptic, Structural coloration, Chromatophore unit

## Abstract

**Background:**

Colour polymorphism in aposematic species is often associated with ecological divergence and predator selection. In amphibians, colouration is achieved through the chromatophore unit, a layered structure of pigment-containing cells (xanthophores, melanophores) and structural cells (iridophores) that interact to produce the final skin colouration. In dendrobatid frogs of the genus *Oophaga*, striking red and green morphs coexist within and across species, yet the cellular mechanisms underlying this variation remain poorly understood.

**Methods:**

We investigated the histological basis of colouration in three *Oophaga* species (*O. granulifera*, *O. pumilio*, and *O. vicentei*) by comparing chromatophore layer structure in red and green morphs and correlating these traits with spectrometric colour measurements. While all species have a chromatophore unit of xanthophores, iridophores, and melanophores, we observed interspecific and intraspecific variation in the organization, thickness, and relative area of these layers.

**Results:**

In *O. pumilio* and *O. vicentei*, red morphs consistently exhibited greater xanthophore and iridophore coverage and more xanthophore contribution. In contrast, *O. granulifera* displayed minimal differences between morphs and a more disorganized chromatophore unit, however melanosome size was larger for green morphs. Spectral reflectance measurements correlated significantly with chromatophore traits, supporting a link between skin histology and colour phenotype. These findings suggest that colouration in *Oophaga* arises from less conserved chromatophore unit structure, reflecting diverse evolutionary routes to equivalent colouration.

## Introduction

Animal colouration plays a crucial role in fitness by mediating communication, mate choice, thermoregulation and interactions with predators (vonHoldt, Bailey & Eizirik, 2021). In visually oriented systems, colour traits function in anti-predator defence through two contrasting strategies, signalling unprofitability and avoiding detection. Aposematic species employ chemical or other defensive strategies and signal their unprofitability as prey through bright, conspicuous colouration (Ruxton et al., 2018). In contrast, less conspicuous species rely on cryptic colouration to avoid detection by blending into their surroundings (Endler, 2006; Ruxton et al., 2018). In some taxa, aposematism and crypsis coexist either across different populations or within the same population (Endler et al., 1997). This results in colour polytypism, which refers to consistent differences in colouration among populations of a species, or colour polymorphism, which describes the presence of multiple colour morphs within a single population (Ford, 1945; Hammond, 1991). These patterns of colour variation are associated with differences in the cellular composition of colour-related skin cells, reflecting the adaptive responses to different selection pressures (Grether, Kolluru & Nersissian, 2007).

Colouration in animals (or plants) can arise through multiple mechanisms, with pigment-based coloration representing a primary, but not exclusive, means of colour production in many taxa (Cuthill et al., 2017). Pigments are chemical compounds that produce colour by selectively absorbing specific wavelengths of light while reflecting others, thereby determining the spectral properties of the colour perceived by observers (Kratochwil & Mallarino, 2023). In mammals, melanin synthesized in melanocytes is the primary pigment responsible for hair and skin coloration (Hearing & Tsukamoto, 1991). Eumelanin produces darker brown to black hues, whereas pheomelanin produces red and yellow hues. In other taxa, coloration often results from the combined effects of pigments and structural coloration mechanisms, as well as the spatial distribution of specialized chromatophore cells (Shawkey & D’Alba, 2017; Gould, 2025).

In amphibians, fish, and reptiles, colouration is produced by three principal types of chromatophore cells, the xanthophores and the melanophores, which contain pigments, and the iridophores, which produce colour through structural mechanisms (Bagnara, Taylor & Hadley, 1968; Grether, Kolluru & Nersissian, 2007; Sköld, Aspengren & Wallin, 2013; Gould, 2025). These chromatophores are primarily found within the dermis, although they may also occur in other skin regions (Bagnara, Taylor & Hadley, 1968; Gould, 2025). Together, these cells are typically arranged in stacked layers that form the dermal chromatophore unit (Bagnara, Taylor & Hadley, 1968).

The outermost layer of the dermal chromatophore unit is composed of xanthophores, which contain carotenoid vesicles and pterinosomes, organelles enriched in carotenoids and pteridines, respectively (Bagnara, Taylor & Hadley, 1968). These pigments selectively absorb and reflect light to produce yellow, orange, and red hues (Bagnara, Taylor & Hadley, 1968; Frost, Epp & Robinson, 1984; Twomey et al., 2020). Beneath the xanthophore layer lies the iridophore layer. Iridophores contain platelet-like crystals composed primarily of purines such as guanine, which produce structural colours through both coherent and incoherent light scattering (Taylor & Bagnara, 1972; Shawkey et al., 2003; Gould, 2025). Iridophores are commonly associated with blue and iridescent hues, contribute strongly to colour brightness, and play a key role in the production of green skin colour when combined with overlying xanthophores and underlying melanophores (Shawkey & D’Alba, 2017; Gould, 2025); however, structural colouration can reflect a broad range of wavelengths depending on platelet size, spacing, and orientation (Shawkey & D’Alba, 2017; Gould, 2025).

The basal layer of the dermal chromatophore unit consists of melanophores, which contain melanosomes, the organelles responsible for melanin synthesis (Bagnara, Taylor & Hadley, 1968). Melanophores influence both hue and brightness by absorbing light and darkening the overlying chromatophore layers, and in regions where melanophores occur alone, they can produce uniformly dark brown or black skin coloration (Bagnara, Taylor & Hadley, 1968; Gould, 2025).

Variation in the density, distribution and arrangement of the chromatophore layers contributes to the final skin colouration and colour variation between morphs has been associated with variation in chromatophore distribution (Bagnara, Taylor & Hadley, 1968; Grether, Kolluru & Nersissian, 2007). In the mimic poison frog, *Ranitomeya imitator*, orange skin tissue is associated with a higher xanthophores to melanophore ratio, compared to green skin tissue where the opposite pattern is observed (de Araujo Miles et al., 2023). Differences in the pigment-based chromatophores present and their density, as well as the size, shape, and arrangement of guanine crystals within iridophores, have been associated with colour variation in *R. imitator* (Twomey et al., 2020). In the harlequin poison frog, *Oophaga histrionica*, colour pattern variation between morphs was associated with differences in the cellular arrangements of the chromatophore unit (Posso-Terranova & Andrés, 2017). Together, these findings highlight that animal colouration is a complex trait emerging from the interactions among multiple chromatophore cell types and their spatial organization within the skin, rather than from variation in any single cell type alone.

The Dendrobatidae family of poison frogs offers many opportunities to study colour variation between populations. In the genus *Oophaga*, species can be polymorphic, polytypic, or in some cases exhibit both, with colour variations between populations associated with differences in predation pressure and adaptive responses to this pressure (Maan & Cummings, 2012; Willink et al., 2013; Dreher, Cummings & Pröhl, 2015). These species generally exhibit two recurrent contrasting colour strategies a red aposematic morph signalling unpalatability and a green cryptic morph that reduces detectability.

The strawberry poison frog, *Oophaga pumilio*, ranging from Nicaragua to Panama, exhibits both polytypism across the Bocas del Toro archipelago and polymorphism on some islands. However, the colour variation most characteristic of the archipelago is the pronounced polytypic pattern, with red, orange, green, and blue morphs occurring across different island populations, and more conspicuous morphs being more unpalatable (Maan & Cummings, 2012). Its sister clade species, *O. granulifera* ranging in the Costa Rica Pacific slope exhibits a red to green gradient in colouration with red frogs in the south, green in the north and intermediate morphs in between (Willink et al., 2013). In both species, the red (aposematic) morph is the ancestral state, while the green (cryptic) morph and other colour morphs have evolved more recently in response to local selection (Brown et al., 2010; Brusa et al., 2013). A lesser-known species of this genus, *O. vicentei*, native to the Caribbean lowlands of Panama is polytypic, with morphs ranging from bright red to dark brown (Lötters et al., 2008; Monteiro et al., 2023). Recent genetic work, places *O. vicentei* close to *O. pumilio* suggesting shared ancestry of two distinct evolutionary lineages (Monteiro et al., 2023).

Despite evidence linking the arrangement and presence of chromatophore types to colour variation in aposematic frogs (Twomey et al., 2020; de Araujo Miles et al., 2023; Monteiro et al., 2025), it remains unclear whether phenotypically similar colour morphs across different species share comparable dermal chromatophore unit composition and layer organization, or whether they achieve similar appearances through different combinations and spatial arrangements of chromatophore types.

Here, we address this gap by comparing red (aposematic) and green (cryptic) morphs across three *Oophaga* species that exhibit visually equivalent colouration. Specifically, we examine whether differences between colour morphs arise from shared *versus* species-specific variation in the relative contribution, thickness, and organization of xanthophore, iridophore, and melanophore layers. By combining histological analyses with spectrometric colour measurements, we assess how variation in dermal chromatophore units underlies repeated evolutionary solutions to respond to predation pressure, resulting in convergent aposematic or cryptic phenotypes across species.

## Methods

### Sampling

Fieldwork was conducted in Costa Rica and Panama during the rainy season in 2022 and 2023. Male individuals of red and green morphs of *Oophaga granulifera*, *O. pumilio* and *O. vicentei* were sampled. Only red and green morphs were selected to represent the most conspicuous and cryptic colour expressions across the three species. All sampled individuals where adult males as sexual dimorphism in dorsal colouration is absent in all three species and because of easier detection due to vocalizing behaviour (Lötters et al., 2008). Additionally, since females are the limiting sex in reproduction (Pröhl & Hödl, 1999) sampling males is less invasive at the population level. This study was conducted in accordance with the Animals in Research: Reporting On Wildlife (ARROW) guidelines for reporting wildlife research. Institutional animal care approval was obtained from the Animal Care and Use Committee (IACUC) of the Smithsonian Tropical Research Institute (STRI; protocol SI-21017). Field sampling and euthanasia procedures complied with national animal welfare legislation and wildlife regulations in Costa Rica and Panama, as authorized by collection permits issued by the Comisión Institucional de Biodiversidad, University of Costa Rica (Resolution No. 331), and the Ministerio de Ambiente de la República de Panamá (permit ARBG-0027-2022).

A total of 80 adult males were sampled across all species and localities, with sample sizes per species and site specified below; numbers were chosen to enable comparisons among colour morphs while minimizing impacts on wild populations. Frogs were captured directly from the field and were not housed in captivity; following capture, individuals were held briefly (<2 h) in individual breathable bags with moist leaf litter to maintain humidity. No feeding, long-term housing, or enrichment was required.

Procedures consisted of non-invasive dorsal skin reflectance measurements followed by terminal tissue sampling. No anesthesia or analgesia was administered during reflectance measurements because procedures were brief, non-invasive, and did not involve tissue injury. Animals were euthanized by immersion in MS-222 followed by double pithing prior to tissue collection. All animals were euthanized as part of the planned experimental design; therefore, no animals were released or maintained after completion of the study.

**Figure 1:**
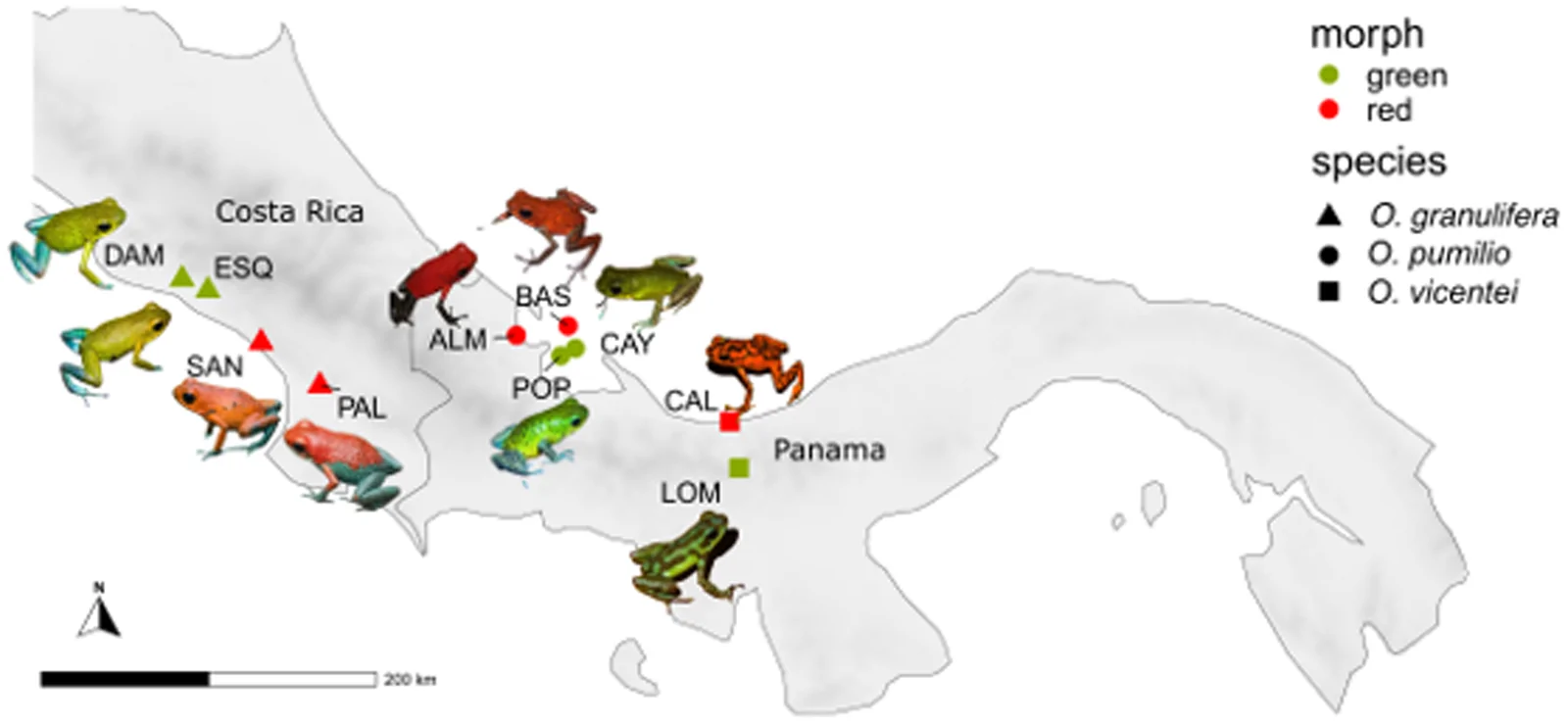
Sampling localities in Costa Rica and Panama. Sampling localities in Costa Rica and Panama where red and green frogs of *Oophaga granulifera* (PAL, SAN, DAM, ESQ), *O. pumilio* (ALM, BAS, POP, CAY) and *O. vicentei* (CAL, LOM) were sampled for extracting skin tissue for histological preparation and analysis. Localities on the map are shown with the code name of each locality, shape indicates the species sampled in the specific locality, colour represents the morph phenotype in each locality. A representative picture of individuals sampled in each locality is added. Photos were taken by the authors AR and RI.

In Costa Rica, green *O. granulifera* frogs were sampled from the northern edge of the species range and red frogs from the southern edge of the species range. Green frogs were collected at Hacienda Mil Bellezas close to Damitas (DAM; N 9.54°, W 84.14°; *N* = 8; Fig. 1) and Esquipulas de Quepos (ESQ; N 9.49°, W 84.05°; *N* = 8), both located in Puntarenas Province. Red morphs were sampled further south at Dominical in San Josecito (SAN; N 9.20°; W 83.75°; *N* = 8) and Palmar Norte (PAL; N 8.97°, W 83.43°; *N* = 8). *Oophaga pumilio* frogs were sampled in Bocas del Toro archipelago in Panama. Red frogs were sampled in the mainland at Valle Agua Arriba, Almirante (ALM; N 9.24°, W 82.08°; *N* = 8; Fig. 1) and in Punta Vieja on Isla Bastimentos (BAS; N 9.29°, W 82.36°; *N* = 8). Green *O. pumilio* were sampled on Isla Cayo de Agua (CAY; N 9.17°, W 82.05°; *N* = 8) and Isla Popa (POP; N 9.14°, W 82.12°; *N* = 8). *Oophaga vicentei* were sampled in the provinces of Colón and Veraguas in Panama. Red frogs were collected in Calovébora (CAL; N 8.79°, W 81.21°; *N* = 4; Fig. 1) and green frogs were sampled in Loma Grande (LOM; N 8.541°, W 81.157°; *N* = 4).

### Spectral measurements

We measured dorsal skin reflectance of the sampled frogs using an Ocean Optics HR2000 spectrometer with a deuterium-tungsten DT-Mini-2-GS lamp and an R-200-2-UV/Vis fiber optic probe (Ocean Optics Inc., Orlando, FL, USA). Before each measurement, the system was calibrated using a WS-1-SS white standard. Reflectance was recorded at four dorsal points in a zig-zag pattern using a dark cylindrical adapter to maintain a two mm distance between the probe and the skin, all under low-light conditions. Spectra were exported *via* OceanView 2.0.10 software (Ocean Optics Inc., Orlando, FL, USA) and processed in R (v4.4.3; R Core Team, 2024) using the *pavo* package (v2.0; Maia et al., 2019). Spectra were trimmed to 300–700 nm, smoothed (0.2 filter), and any negative values were set to zero. Individual reflectance curves were averaged and summarized into brightness (B1–B3), chroma (S1–S10), and hue (H1–H5) descriptors using *pavo*’s summary function (Montgomerie, 2006). To assess how distinguishable red and green morphs are to a visual predator across species, we modelled avian vision using a tetrachromatic UV-sensitive bird visual system. We calculated just-noticeable differences (JNDs) between morphs of each species and visualized these colour distances in perceptually corrected Cartesian space using the *pavo* package (Maia et al., 2019).

### Tissue sampling, bright field and transmission electron microscopy

A section of dorsal skin from the nuchal region was excised and fixed in 10% neutral-buffered formalin for histological processing. Samples were stored in accredited laboratories in Costa Rica and Panama for up to six months pending permit approval, after which they were transported to the Histology Laboratory in Germany for further processing. Fixed tissues were post-fixed in 1% osmium tetroxide, dehydrated through an ethanol series, and embedded in Epon 812 resin. Semithin sections (1–2 µm) were cut using an ultramicrotome, stained with toluidine blue, and imaged using a Zeiss Axioplan 2 light microscope (Zeiss, Oberkochen, Germany) at 100 ×, 200 ×, and 400 × magnification.

Quantitative image analysis was conducted in ImageJ (v1.51q; Schneider, Rasband & Eliceiri, 2012). For each individual, six high-resolution images (1,920 ×1,200 pixels) were captured at 400 × magnification using a DFK 23UX249 camera (The Imaging Source, Bremen, Germany) and IC Capture 2.4 software (The Imaging Source, Bremen, Germany). Spatial calibration was performed using a Thoma hemocytometer (2.5 µm grid) to convert pixel measurements to micrometers. Brightness, contrast, and reference levels were standardized across all images prior to analysis.

In ImageJ, using the 400 × images, we measured the area coverage of each chromatophore layer with the free-hand selection tool. The tissue area was defined as the entire dermal layer visible in the histological section, and the chromatophore layer area was quantified as the portion of this dermal tissue area that was covered by cells of the respective chromatophore type. When chromatophores of the same type were spatially discontinuous, then the area of each cell present was summed. The thickness of each chromatophore layer (*i.e.,* the vertical distance from the top to the bottom of that layer), the total tissue thickness, and the thickness of the collagen-rich compact dermis (hereafter refered to as the collagen layer) were measured within the dermal layer using the straight-line tool (see SupMat1; Fig. SA1). The collagen layer forms the dermal region directly beneath the chromatophores and was included in the total tissue thickness measurement. Measurements were transformed to micrometers using the Thoma hemocytometer conversion factor (1 pixel = 0.01217 µm) and subsequently adjusted to account for total tissue thickness. Layer thickness was determined by averaging three independent measurements per image. For each individual, measurements were taken from six independent images, and the resulting values were averaged. Images exhibiting substantial degradation of cellular structure were excluded from the analysis (see SupMat1; Fig. SA2).

To compare across individuals with different absolute skin sizes, we calculated the relative contribution of each chromatophore type to the total chromatophore unit. For each sample, the total chromatophore unit area was calculated as the sum of the xanthophore, melanophore, and iridophore areas. This approach standardizes chromatophore contribution by expressing each layer as a proportion of the entire chromatophore unit, allowing comparisons across species and morphs independent of absolute tissue size or total chromatophore area.

Ultrathin skin sections were prepared for transmission electron microscopy (TEM) using standard fixation, dehydration, and resin-embedding procedures. Sections from three individuals per population were imaged on a Zeiss LEO 906 TEM at multiple nominal magnifications (×2,156; ×3,597; ×4,646; ×6,000; ×7,750; ×16,700). For quantitative analyses, images acquired at ×6,000, ×7,750 and ×16,700 were used. For each individual and each of these magnifications, six non-overlapping fields of view were captured and analysed.

Using ImageJ (Schneider, Rasband & Eliceiri, 2012), the spatial calibration for each TEM image was set from the embedded scale bar using the scale tool of ImageJ (Analyze >Set Scale) to convert pixel distances to micrometers (µm) prior to each measurement. In ImageJ, using the straight-line tool, we took measurements of the melanosome size by selecting eight melanosomes per image and averaged melanosome size per image and then per individual across the six images. For xanthophores, the sizes of carotenoid vesicles and pterinosomes were measured in the samme manner (see Fig. SA3 for examples of carotenoid vesicles and pterinosomes). Melanosome density in the melanophores was quantified using a custom python script. Images were converted to greyscale and thresholded (pixel intensity <50) to segment melanosomes which are circular black organelles. Each melanosome was detected as a separate organelle (if two or more melanosomes were connected and edges were not detected these were assumed as degraded organelles and therefore excluded), and the total count per image was recorded. Melanosome density was expressed as the number of melanosomes per µm^2^.

### Statistical analysis of chromatophore layer variation between morphs and species

To compare histological characteristics between red and green morphs within each species, we used the Mann–Whitney U test (Mann & Whitney, 1947). This nonparametric test does not assume normality and is appropriate for comparisons between two independent groups. Analyses were conducted separately for each species and histological variable. Variables were excluded when all observations were missing or when only one morph was represented. Tests were implemented in R (v4.4.3; R Core Team, 2024) using the wilcox.test() function with independent samples (paired = FALSE). For each comparison, the U statistic and corresponding pvalue were calculated. Summary statistics are reported as medians and interquartile ranges and are presented on the original measurement scale.

### Statistical analysis of correlation between spectral measurements and tissue

To identify spectral variables that best explain the variation between red and green morphs across the three species a principal component analysis (PCA) was conducted on the summarized spectral variables. Selected spectral variables were used in a canonical correlation analysis (CCA) to investigate the relationships between spectral variables to histological variables. The response matrix (Y) consisted of non-redundant spectral variables that contribute the most to observed colour variation between morphs including, intensity (B3: Maximum relative reflectance, Reflectance at wavelength of maximum reflectance), carotenoid chroma (S9: reflectance due to carotenoid pigmentation), relative contribution of a spectral range to the total brightness (S1V: violet, 300 nm–415 nm, S1G: green, 510 nm–605 nm). The explanatory matrix (X) included 11 histological traits including chromatophore layer area coverage and thickness. To examine relationships between spectral reflectance and histological traits, we performed pairwise Pearson correlation tests separately for red and green colour morphs using base R functions. Only individuals with complete data for both spectral and histological variables were included in the analysis. For each morph, Pearson correlation coefficients (r) and corresponding two-tailed *p*-values were computed using the cor() and cor.test() functions in base R (v4.4.3; R Core Team, 2024). The *p*-values were derived under the assumption of bivariate normality, using the t-distribution to test the null hypothesis that the correlation coefficient is zero. Correlation tests were conducted between each spectral variable (B3, S9, S1V, S1G) and each histological variable. Statistically and biologically relevant correlations were defined as those with —r— ≥ 0.4 and *p* ≤ 0.05.

## Results

### Visual perception of red and green morphs

**Figure 2:**
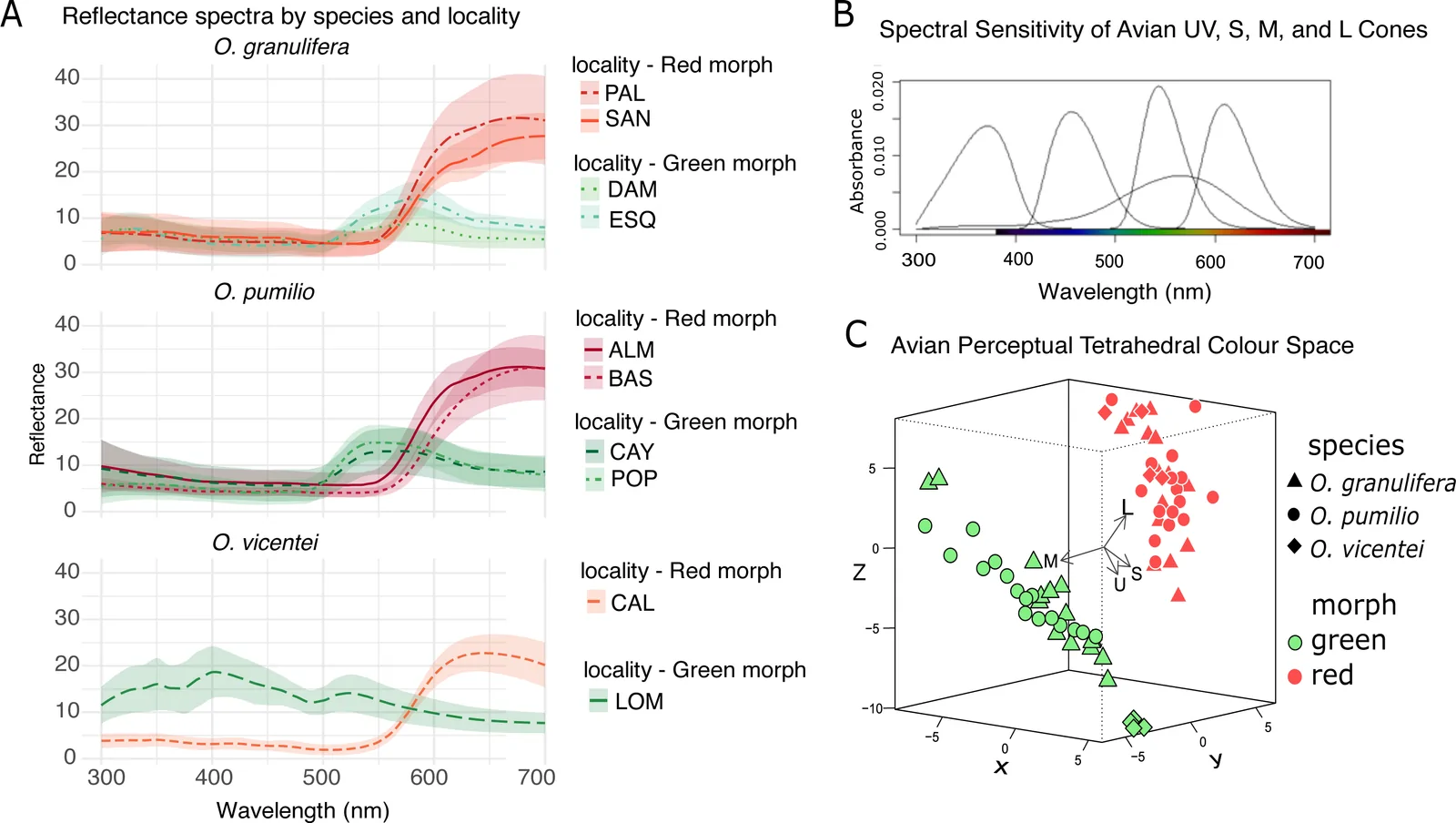
Spectral reflectance and avian visual modelling of colour morphs in three *Oophaga* species. (A) Reflectance spectra for red and green morphs of *O. granulifera*, O*. pumilio*, and *O. vicentei*, shown separately by species and morph. Bold lines represent mean reflectance per locality, and thin lines show individual frogs. Line types distinguish sampling localities, and colours indicate morph type. (B) Avian tetrachromatic UV sensitive visual system modelled. (C) Avian tetrahedral colour space showing perceptual clustering of red and green morphs across species. The tetrahedral space represents relative stimulation of the four avian cone types (L, M, S, U) after normalisation, which constrains tetrahedral chromaticity to three dimensions despite tetrachromatic input.

Reflectance spectra differed consistently between red and green frogs across all three *Oophaga* species (Fig. 2A). Although all red morphs fall within a red to red-orange range, they show slight differences in hue among species: *O. pumilio* exhibits a saturated bright red, *O. granulifera* shows a deeper red-orange tone, and *O. vicentei* displays a somewhat duller red. Green morphs also vary visibly across species, ranging from a vivid green in *O. pumilio* to a yellow-green in *O. granulifera* and a more muted bluish-green in *O. vicentei*. Red frogs showed higher reflectance at longer wavelengths (peaking in red region, ∼600–700 nm) and comparatively low reflectance at shorter wavelengths. In contrast, green frogs showed higher reflectance at shorter to intermediate wavelengths, particularly within the green spectral range (∼500–600 nm), with lower reflectance at longer wavelengths. Visual modelling revealed that red frogs of the three species cluster closely in avian perceptual colour space (Fig. 2C). Green morphs of *O. granulifera* and *O. pumilio* closely cluster together, while the four green frogs of *O. vicentei* appear to cluster together slightly separated from the other green frogs.

### Interspecific chromatophore differences

**Figure 3:**
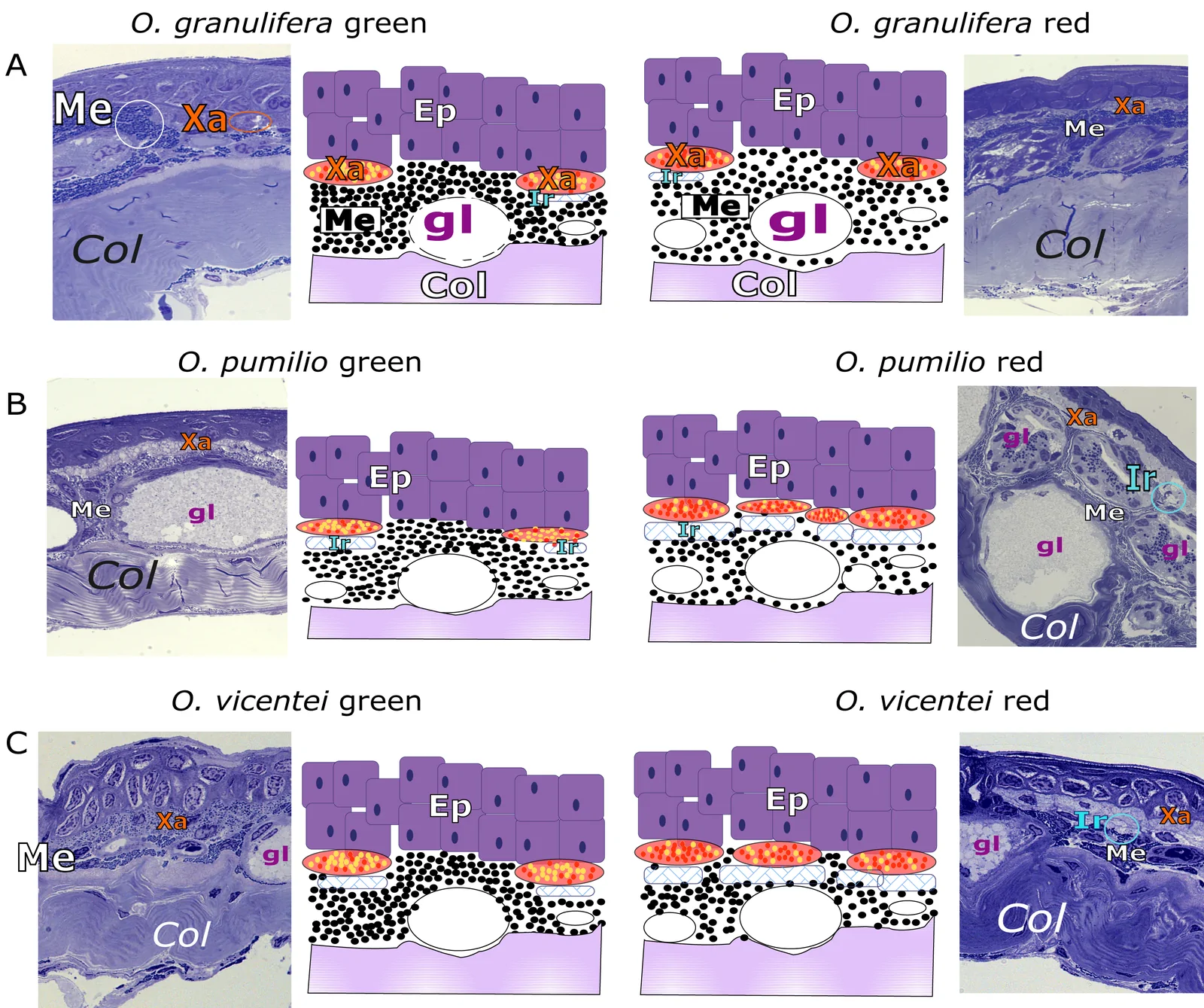
Descriptive overview of the chromatophore unit organization differences among the three *Oophaga* species and between red and green morphs. (A) *O. granulifera* green and red morphs. Representative bright-field images of green (left) and red (right) individuals taken at 400x magnification are shown. Central schematic illustration representing the key morphological differences between the morphs. (B) *O. pumilio* green and red morphs, with representative images and schematics. (C) *O. vicentei* green and red morphs, with representative images and schematics. Abbreviations: Me, Melanophore layer; Xa, Xanthophore layer; Col, Collagen layer; Ep, Epidermis; gl, glands; Ir, Iridophore layer.

Histological analysis of the dorsal skin revealed interspecific differences in the organization of the dermal chromatophore unit that varied between red and green morphs (Fig. 3).

Among red morphs, *O. granulifera* exhibited a less consistently stratified chromatophore unit compared to *O. pumilio* and *O. vicentei* (Fig. 3). In *O. granulifera* red individuals, a xanthophore (Xa.) layer was present directly beneath the epidermis (Ep.) but was often discontinuous and irregular across individuals. In these frogs, melanophores (Me.) were typically located immediately below the xanthophores, while iridophores (Ir.) were present only sparsely. When iridophores were present, they occupied the intermediate position between the xanthophore and melanophore layers, and the melanophores frequently wrapped around or partially enclosed these isolated iridophore cells. In many red *O. granulifera* frogs, the iridophore layer was completely missing.

In contrast, red frogs of *O. pumilio* and *O. vicentei* showed a more clearly stratified chromatophore unit, with xanthophores forming a continuous superficial layer, a distinct iridophore layer beneath, and melanophores occupying the basal position (Fig. 3).

Among green frogs, similar interspecific differences were observed. In *O. granulifera,* green frogs showed a comparatively less organized chromatophore unit, characterized by irregular xanthophore coverage and largely absent or discontinuous iridophore layer. By contrast, green frogs of *O. pumilio* and *O. vicentei* retained a layered arrangement of chromatophores, although with differences in relative layer thickness and coverage between species.

Across species and morphs, vertical associations between chromatophore layers were variable. Melanophores were often observed without xanthophores or iridophores directly overlying them; however, we never observed chromatophore units composed solely of xanthophores or iridophores without melanophores. In *O. granulifera*, iridophores were present only in a small number of individuals. In green *O. vicentei*, melanophores were frequently exposed without superficial xanthophores or iridophores, whereas red *O. vicentei* individuals almost always showed a complete vertical sequence of xanthophores, iridophores, and melanophores, similar to the pattern consistently observed in red *O. pumilio*.

These patterns highlight clear species-level differences in chromatophore organization. *Oophaga pumilio* and *O. vicentei* typically show well-defined layers, whereas *O. granulifera* is more variable. Glandular structures were found in all species, usually positioned between chromatophore layers and adjacent to the compact dermis (stratum compactum). Within species, red and green morphs differed in chromatophore composition and organization, but no single pattern of morph-specific variation was shared across all species.

### Intraspecific variation in chromatophore layers and cells

**Figure 4:**
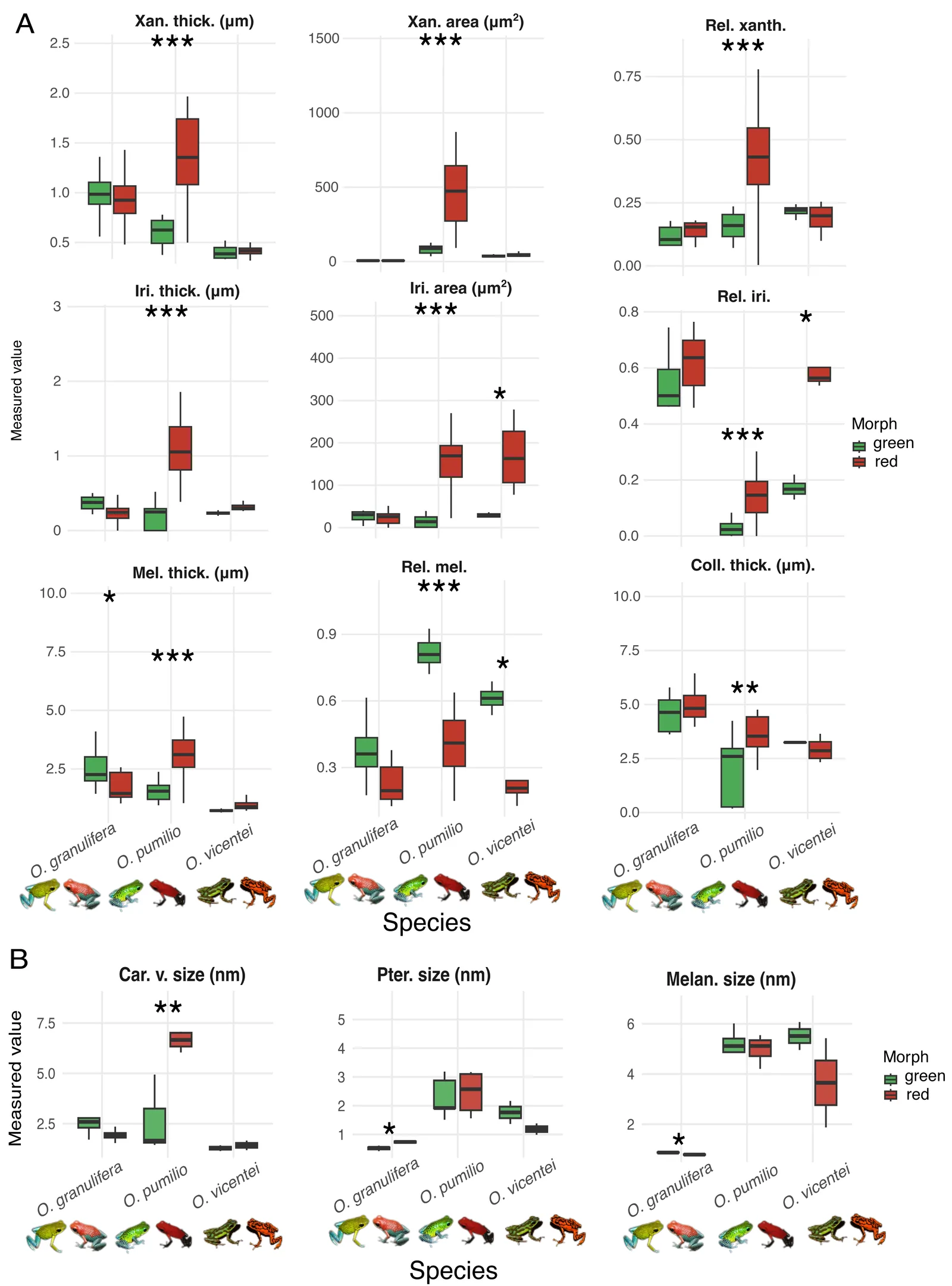
Comparison of significant histological and ultrastructural traits between red and green morphs across three *Oophaga* species. (A) Histological variables measured from brightfield microscopy, including chromatophore layer thickness (Coll. thick., Mel. thick., Xan. thick., Iri. thick.), chromatophore area (Mel. area, Xan. area, Iri. area), and relative chromatophore layer contributions (Rel. xanth., Rel. mel., Rel. iri.). Each panel displays a single variable, plotted across species (*O. granulifera*, *O. pumilio*, *O. vicentei*) and morphs (green, red). (B) Ultrastructural variables measured from transmission electron microscopy, including carotenoid vesicle size (Car. *v.* size), pteridosome size (Pter. size), and melanosome size (Melan. size). Each variable is shown in a separate panel with its corresponding units. Asterisks indicate statistically significant differences between morphs (Wilcoxon ranksum test; *p < 0.05, **p < 0.01, ***p < 0.001). Photos were taken by the authors AR and RI.

Histological comparisons of iridophore, melanophore, and xanthophore layers revealed species-specific differences in layer area coverage and thickness (Fig. 4).

Pairwise U-tests (Fig. 4; see Table SM2 for detailed measurements and Table SM3 for full U-test results) revealed multiple significant differences in chromatophore structure between morphs within species. In *O. granulifera,* green frogs had a thicker melanophore layer than red frogs and the number of glands was significantly higher for red frogs. No other significant variation was observed between morphs of *O. granulifera.* In *O. pumilio*, red frogs consistently showed higher values across several chromatophore traits. Red individuals had a thicker collagen layer, greater xanthophore and iridophore layer thickness, and larger xanthophore and iridophore area coverage. Red frogs also exhibited higher relative xanthophore and relative iridophore contributions compared to green frogs. No significant differences were detected for melanophore area or melanophore thickness in this species*.* In *O. vicentei*, significant variations between morphs were observed in iridophore area coverage, which was greater for red frogs. Relative iridophore contribution differed significantly between morphs, with red frogs showing higher values than green frogs, while relative melanophore contribution was higher for green frogs. All other histological variables showed no significant morph-specific differences in this species. Among the chromatophore traits examined, melanophore layer thickness differed significantly between morphs in *O. granulifera* and *O. pumilio* and the two species showed opposite trends. Red *O. pumilio* had a thicker melanophore layer than green individuals of this species, whereas green *O. granulifera* had a thicker melanophore layer than red individuals of this species. Across species, xanthophore- and iridophore-related traits (layer area, layer thickness, and relative contribution) were consistently higher in red *O. pumilio*, whereas *O. granulifera* and *O. vicentei* showed more limited morph-specific variation.

### Variation in organelle sizes inside chromatophore cells

**Figure 5:**
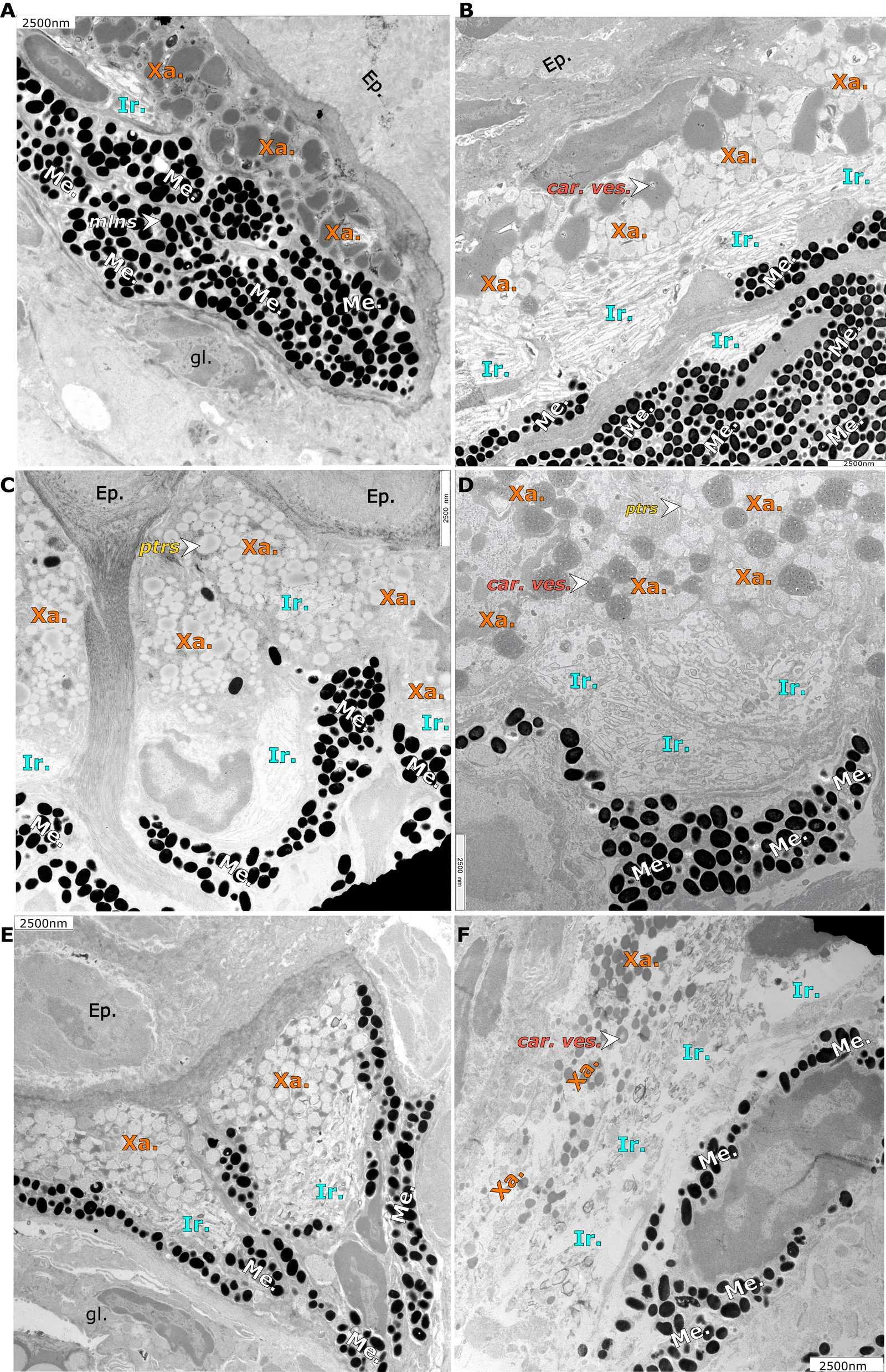
Transmission electron microscopy (TEM) images of chromatophores of three *Oophaga* species and associated cellular structures measured using TEM images. (A–B) *Oophaga granulifera*, green (A) and red (B) morph. (C–D) *Oophaga pumilio* green (C) and red (D) morph. (E–F). *Oophaga vicentei* green (E) and red (F) morph. Identified cellular structures include: Xa., xanthophores; Me., melanophores; Ir., iridophores; Ep., epidermis; gl., glands; mlns., melanosomes; ptrs., pterinosomes; car. ves., carotenoid vesicles. Image illustrates the chromatophore unit layered structure contributing to colour expression of the skin in each morph. All images were taken from the first author (VMO).

Transmission electron microscopy revealed a few differences in chromatophore layer structure and organization between green and red colour morphs across the three *Oophaga* species (Fig. 4B; see Table SM3 for U test results; Table SM4 for full measurements). At a descriptive level, green morphs consistently exhibited a chromatophore unit with dense melanophores, rich in melanosomes. Xanthophores containing pterinosomes and carotenoid vesicles were visible. Pterinosomes appeared as circular organelles within xanthophore cells and contained irregular grey dots or circular structures in TEM images (Fig. 5). Carotenoid vesicles were membrane-bound organelles in xanthophore cells that appeared as grey circular structures containing small whitish dots in TEM images (Fig. 5). In *O. pumilio* and *O. granulifera*, the extracellular matrix (ECM) was highly visible and appeared prominently between chromatophore units (Fig. 5).

In *O. granulifera*, melanosome size within the melanophore layer was significantly larger in green frogs (Fig. 4B; *p* = 0.018; see Table SM3), while pterinosomes within the xanthophore layer were significantly larger in red frogs (Fig. 4B; *p* = 0.019; see Table SM3).

In *O. pumilio*, no significant differences were detected between red and green frogs in melanosome density or melanosome size, both measured within the melanophore layer, nor in pterinosome size, measured within the xanthophore layer (Fig. 4B; see Table SM3). In contrast, carotenoid vesicles within the xanthophore layer were significantly larger in red frogs (Fig. 4B; *p* = 0.030; see Table SM3).

No significant differences were observed between red and green frogs of *O. vicentei* for any variables measured from TEM images. Although the size and precise orientation of iridophore reflecting platelets could not be quantified due to insufficient preservation, the available images (*e.g.*, Fig. 5B) show that platelets were generally arranged in parallel layers aligned with the skin surface. In some individuals, melanophores extended upward through the dermal chromatophore unit; for example, in Fig. 5E, melanophore processes reach into higher layers and partially surround both the iridophore and xanthophore layers, a pattern not observed in the other species.

### Correlation between chromatophore cells and colouration

**Figure 6:**
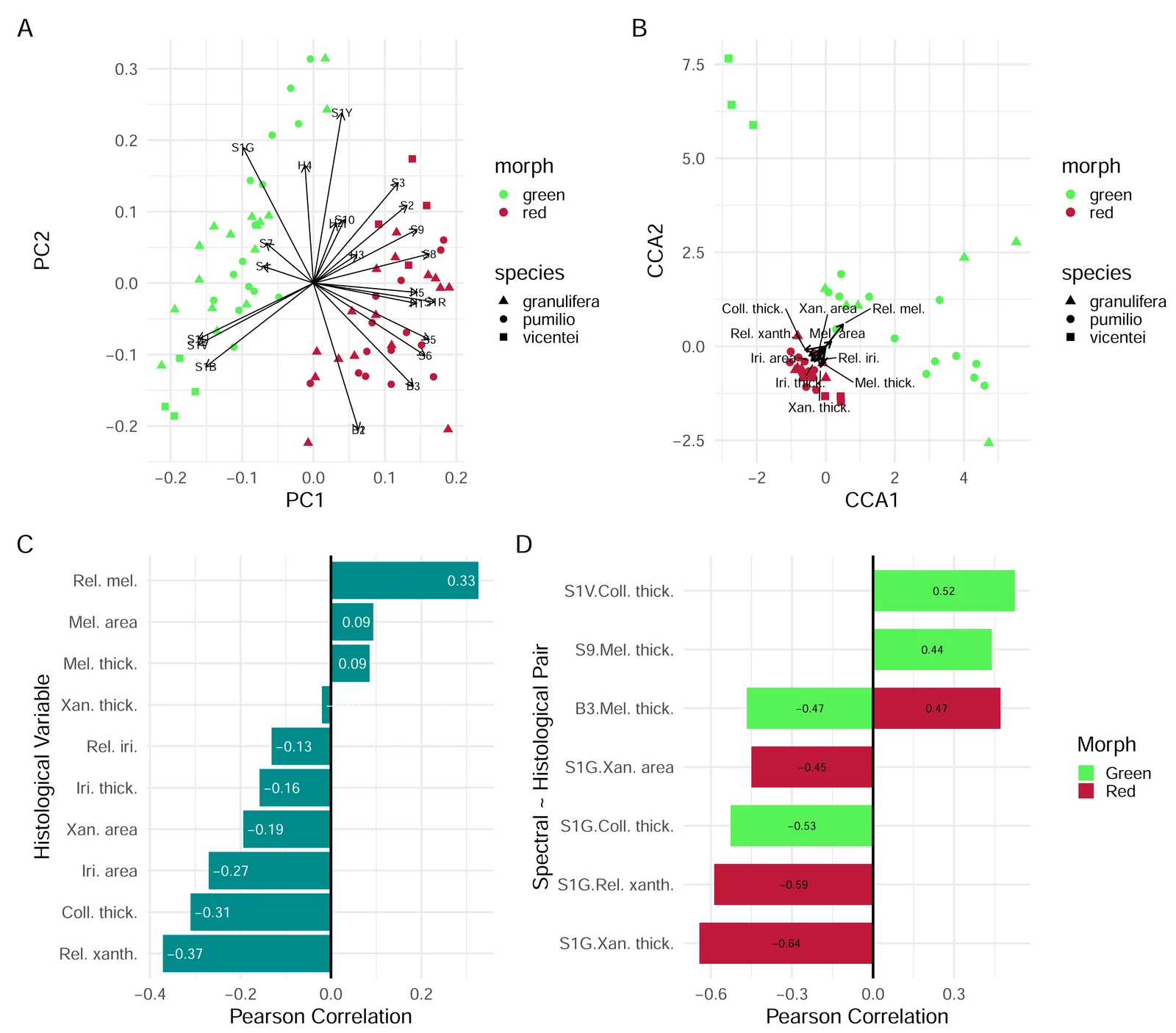
Multivariate analyses linking spectral reflectance and histological traits across morphs and species. (A) Principal Component Analysis (PCA) of spectral reflectance variables. Colour indicates morph (red or green), and point shape indicates species. (B) Canonical Correspondence Analysis (CCA) biplot showing the association between spectral variables and histological traits. Arrows represent histological variables contributing to variation across morphs. Colour indicates morph, and point shape indicates species. (C) Loadings of histological variables on the first canonical axis (CCA1), illustrating the relative contribution of each trait to the observed spectral variation. (D) Significant Pearson correlations between spectral and histological variables. Bars represent correlation coefficients and are coloured according to the morph in which each correlation was detected (red or green). Only correlations passing the significance threshold (BH adjusted *p* < 0.1) are shown.

A CCA between the non-redundant spectral variables as the response matrix and histological variables as the explanatory variables showed that the histological variable with the strongest loading on CCA1 were the relative contributions of each chromatophore layer (Fig. 6; Table SM5). Other key contributors included xanthophore layer thickness, collagen layer thickness, xanthophore area, and melanophore area.

Pearson correlation analysis between spectral and histological variables showed morph-specific relationships between histological and spectral traits. In red morphs, significant correlations were found between S1G and several xanthophore-related traits, including xanthophore thickness (*r* = −0.64, *p* < 0.001), relative xanthophore contribution (*r* = −0.59, *p* = 0.002), and xanthophore area (*r* = −0.45, *p* = 0.027). Red morphs also showed a positive correlation between B3 and melanophore layer thickness (*r* = 0.47, *p* = 0.020).

In green morphs, spectral variation was primarily associated with collagen and melanophore traits. S1V was positively correlated with collagen layer thickness (*r* = 0.52, *p* = 0.012), while S1G showed a negative correlation with collagen thickness (*r* = −0.53, *p* = 0.012). Green morphs also showed significant correlations between melanophore thickness and both B3 (*r* = −0.47, *p* = 0.029) and S9 (*r* = 0.44, *p* = 0.041). No significant correlations were detected between spectral variables and iridophore area or thickness in either morph after multiple-testing correction (full results in SM6).

## Discussion

Our comparative study shows that the red and green colour morphs appear relatively equivalent across *Oophaga granulifera*, *O. pumilio*, and *O. vicentei*, even though the specific hues differ slightly among species and between morphs within species. Avian visual modelling further indicates that predators are unlikely to distinguish between red frogs of the three species, or between green frogs of the three species, suggesting perceptual equivalence of these colour categories. Yet, despite this similarity in perceived colour, the cellular composition and organization of the dermal chromatophore units differ markedly among species and morphs. Overall, our results demonstrate that distinct structural and cellular arrangements can give rise to equivalent colour signals, highlighting how species repeatedly evolve similar aposematic or cryptic phenotypes in response to predation pressure.

Red frogs were generally characterized by increased development of the xanthophore and iridophore layers, observed as greater layer thickness and coverage, which together enhance brightness and chromatic saturation. Red colouration functions as a highly salient aposematic signal, facilitating predator learning and avoidance in poison frogs and other taxa (Summers & Clough, 2001; Siddiqi et al., 2004; Maan & Cummings, 2012). In contrast, green frogs rely on interactions between melanophores and structurally organized iridophores, with green hues arising from structurally reflected blue and green light filtered through the overlying xanthophore layer. Green coloration is commonly associated with crypsis, reducing visual contrast with vegetation (Stevens & Merilaita, 2009). Together, these patterns demonstrate that distinct colour phenotypes are achieved through variation in chromatophore composition and organization rather than through a single conserved cellular signature, consistent with observations in other dendrobatids (de Araujo Miles et al., 2023; Twomey et al., 2020; Posso-Terranova & Andrés, 2017).

At the species level, *O. pumilio* and *O. vicentei* exhibited highly similar and consistently organized chromatophore units, whereas *O. granulifera* displayed a more divergent structure, likely reflecting phylogenetic relationships (Monteiro et al., 2023; Posso-Terranova & Andrés, 2017). Although visual modelling suggests equivalent colour perception by birds across species, the green phenotype of *O. granulifera* is produced with limited iridophore involvement, resulting in a more pigment-based dark yellow–green appearance rather than a bright green. In contrast, green coloration in *O. pumilio* and *O. vicentei* arises primarily from structurally organized iridophores filtered by overlying xanthophores, indicating a shared structural colour mechanism. While iridophore platelet organization could not be quantified, the available TEM images show that reflecting platelets of both *O. pumilio* and *O. vicentei* are layered and generally parallel to the skin surface, an arrangement that is likely to contribute to multilayer interference and the selective reflection of blue–green wavelengths. The similarity in chromatophore layering and relative contributions of pigmentary and structural chromatophores in *O. pumilio* and *O. vicentei* suggests conserved developmental regulation or similar selective pressures, consistent with their close evolutionary relationship. In contrast, both red and green morphs of *O. granulifera* show a reduced contribution of iridophores to colour expression, which may reflect distinct selective pressures on signal design or its more distant evolutionary relationship to *O. pumilio* and *O. vicentei*.

Across *O. granulifera*, *O. pumilio*, and *O. vicentei*, no single chromatophore-related trait consistently distinguished red (aposematic) from green (cryptic) frogs across all three species. However, when *O. granulifera* is excluded, green *O. pumilio* and O*. vicentei* show shared features, including a more organized dermal chromatophore unit with a clearly developed iridophore layer, consistent with structurally mediated green colouration. In both species, the extracellular matrix (ECM) also appeared more prominent in green frogs, suggesting a role in pigment cell positioning and organization. The ECM, a collagen- and protein-rich network, is known to influence pigment cell migration and spatial arrangement through integrin signalling and cytoskeletal dynamics in other vertebrates (Rozario & De Simone, 2010; Parichy, 2006; Kelsh et al., 2009).

Our results reveal strong associations between chromatophore traits and spectral variables, which can be mechanistically linked to colour production. In red frogs, green reflectance (S1G) was negatively correlated with xanthophore thickness, xanthophore area, and relative xanthophore contribution, indicating that thicker and more extensive xanthophore layers absorb more short wavelengths and enhance long wavelength reflectance. Red frogs also showed a positive correlation between B3 (maximum reflectance at the spectral peak) and melanophore thickness, suggesting that variation in melanophore development contributes to brightness and contrast in aposematic signals.

In green frogs, reflectance in the violet and green regions was associated with collagen and melanophore thickness, supporting a predominantly structural basis of green coloration. Collagen thickness was positively correlated with S1V and negatively correlated with S1G, consistent with its role in modulating light scattering and filtering (Prum & Torres, 2003; Bagnara, Fernandez & Fujii, 2007; Saenko et al., 2013).

Structural colours produced by iridophores often arise from coherent light reflection by ordered platelet arrays and are commonly filtered by overlying xanthophore pigments and modulated by underlying melanophores, resulting in green hues without direct green pigment production (Taylor & Bagnara, 1972; Shawkey & D’Alba, 2017; Gould & McHenry, 2024). Although the size, orientation, and nanostructural organisation of individual iridophore platelets were not quantified here, differences in iridophore development and layering are known to influence spectral properties in amphibians and other vertebrates (Bagnara, Taylor & Hadley, 1968; Gould & McHenry, 2024).

Both red and green colouration in *O. pumilio* and *O. vicentei* involve iridophores but contrasting colour phenotypes arise from differences in the relative contributions and spatial arrangement of pigmentary and structural chromatophores. Red aposematic phenotypes are associated with increased pigment filtering by xanthophores and enhanced luminance from reflective layers, whereas green phenotypes rely more strongly on structurally mediated reflectance modified by surrounding chromatophore layers, a pattern consistent with mechanisms described in other dendrobatid frogs (de Araujo Miles et al., 2023; Gould & McHenry, 2024).

Colouration in *Oophaga* is not produced by a single, uniform mechanism but instead arises through different combinations of chromatophore types and their organization within the skin. In red morphs, the iridophore layer likely functions primarily as a broadband reflector that enhances the brightness of overlying xanthophore pigments, whereas in green frogs it selectively reflects blue–green wavelengths and interacts with xanthophores to generate structural green. Further ultrastructural analyses are needed to determine whether platelet arrangement differs between morphs and how these differences contribute to colour production. Although red and green colour phenotypes appear similar across species, they can be generated through distinct cellular and developmental pathways shaped by species history and local ecological pressures (Demski, 2008; Twomey et al., 2023).

##  Supplemental Information

10.7717/peerj.21696/supp-1 Supplemental Information 1 Supplemental figures illustrating histological and ultrastructural measurements Figure A1 (measurement workflow), Figure A2 (image exclusion/quality control), Figure A3 (organelle ultrastructure),

10.7717/peerj.21696/supp-2 Supplemental Information 2 Integrated raw dataset of skin histological measurements and spectral reflectance data for all sampled individuals individual-level information including slide and specimen identifiers, colour morph, locality, and species. Histological variables comprise tissue thickness, collagen layer thickness, chromatophore (iridophore, melanophore, and xanthophore) area, thickness, and density, as well as derived ratios and composite indices. Spectral variables include reflectance values from discrete spectral bands (B- and S-bands) and hyperspectral components (H-bands). All values represent measurements extracted from microscopy images and spectral recordings and form the basis for statistical analyses and multivariate models presented in the main text and supplemental materials.

10.7717/peerj.21696/supp-3 Supplemental Information 3 Results of Mann–Whitney U tests comparing skin ultrastructural variables between red and green colour morphs across species For each variable, median values with interquartile ranges (IQR) are reported for red and green morphs. The Mann–Whitney U statistic and associated p -value are shown, and results are classified as significant at *p* < 0.05. The direction column indicates which colour morph presents higher median values. Variables were quantified using transmission electron microscopy (TEM) or bright-field microscopy (BFM), as indicated. Images measured refers to the microscopy method used for each variable.

10.7717/peerj.21696/supp-4 Supplemental Information 4 Raw transmission electron microscopy (TEM) measurements of chromatophore ultrastructure across colour morphs and species individual samples (slide and ID), colour morph, and species identity, along with quantitative measurements of melanosome density and organelle size metrics, including melanosome length, carotenoid body (carotene vesicle) length, and pterinosome length. All values represent measurements extracted from TEM images and form the basis for summary statistics and non-parametric comparisons presented in the main text and other supplemental materials.

10.7717/peerj.21696/supp-5 Supplemental Information 5 Canonical correspondence analysis (CCA) results linking skin histological variables to spectral reflectance properties canonical coefficients for histological predictors across the first three canonical axes (CCA1–CCA3), as well as structure coefficients (loadings) for histological and spectral variables on the first two canonical axes. Histological variables include chromatophore layer thickness, area, number-area ratios (NAR), composite indices, and gland abundance. Spectral variables correspond to reflectance bands (B3, S9, S1V, S1G). Loadings indicate the relative contribution and direction of each variable to the canonical axes.

10.7717/peerj.21696/supp-6 Supplemental Information 6 Correlations between spectral variables and skin histological traits analysed separately for red and green colour morphs For each morph, bivariate correlations were calculated between spectral measurements (S1–S9, B3) and histological variables using Pearson’s correlation coefficient (r). Reported values include the correlation coefficient (r), associated p-value, Benjamini–Hochberg false discovery rate–adjusted p-value (p _adj), sample size (n), and absolute correlation strength (—r—). Significance indicates correlations passing the predefined threshold after multiple-testing correction, with asterisks denoting nominal significance levels (*p* < 0.05=*, *p* < 0.01=**, *p* < 0.001=***).

## Additional Information and Declarations

### Field Study Permissions

The following information was supplied relating to field study approvals (*i.e.*, approving body and any reference numbers): Collection and fieldwork permits in Costa Rica were issued by the Comisión Institucional de Biodiversidad of the University of Costa Rica (Resolution No. 331). In Panama, the sampling protocol (SI-21017) was approved by the Animal Care and Use Committee (IACUC) of the Smithsonian Tropical Research Institute (STRI), and the collection permit (ARBG-0027-2022) was granted by the Ministerio de Ambiente de la República de Panamá.

### Data Availability

The following information was supplied regarding data availability: The data and code are available at Zenodo: Mantzana Oikonomaki, V. (2026). HISTOVMO [Computer software]. Zenodo. https://doi.org/10.5281/zenodo.20310025.
