# Supplementary material for: Frogs have layers: a comparative histological study of colour polymorphism in Oophaga poison frogs
Source: PeerJ. 2026 Sep 16;14:e21696. doi: 10.7717/peerj.21696 (PMC13589513; doi:10.7717/peerj.21696)
Supplement: Supplementary file 1 [file peerj-14-21696-s001.docx]

Figure A1.: Example of taking measurements of Tissue and layer thickness (A) and coverage (B). (A) From each picture for each individual, 3 measurements of each variable were taken and averaged per image and then per individual. Black shows measurements of whole tissue thickness, pink of collagen layer, white of melanophore layer, blue of iridophore layer and yellow of xanthophore layer. (B) An example of measuring chromatophore layer covrage for xanthophores (yellow overlay), iridophores (blue overlay) and melanophores (black overlay). Whole tissue coverage was also taken to estimate the are of each layer in terms of total tissue area (not shown as an overlay for clarity). Density of each chromatophore layer was calculated by layer coverage adjusted for layer thickness.

Figure A2.: Example of images that were discarded of analysis due to degradation of skin tissue at a large scale. In this example, melanophore layer and xanthophore layer is highly degraded. Histological slides were prepared again for such samples to get a better result when possible. The individual shown here, a new set of histological slides was prepared were chromatophore layers where not degraded

**Figure A3.:** Example of carotenoid vesicles organelles (A) and pterinosomes (C). Size of organelles were measured my estimating the size of 8 organelles per image and averaging per image and sequentially per individual from 6 images.
